# Textronic Glove Translating Polish Sign Language

**DOI:** 10.3390/s22186788

**Published:** 2022-09-08

**Authors:** Ewa Korzeniewska, Marta Kania, Rafał Zawiślak

**Affiliations:** 1 Institute of Electrical Engineering Systems, Lodz University of Technology, Stefanowskiego 18 Street, 90-537 Lodz, Poland; 2 Institute of Automatic Control, Lodz University of Technology, Stefanowskiego 18 Street, 90-537 Lodz, Poland

**Keywords:** textronics, wearable electronics, sign glove

## Abstract

Communication between people is a basic social skill used to exchange information. It is often used for self-express and to meet basic human needs, such as the need for closeness, belonging, and security. This process takes place at different levels, using different means, with specific effects. It generally means a two-way flow of information in the immediate area of contact with another person. When people are communicating using the same language, the flow of information is much easier compared to the situation when two people use two different languages from different language families. The process of social communication with the deaf is difficult as well. It is therefore essential to use modern technologies to facilitate communication with deaf and non-speaking people. This article presents the results of work on a prototype of a glove using textronic elements produced using a physical vacuum deposition process. The signal from the sensors, in the form of resistance changes, is read by the microcontroller, and then it is processed and displayed on a smartphone screen in the form of single letters. During the experiment, 520 letters were signed by each author. The correctness of interpreting the signs was 86.5%. Each letter was recognized within approximately 3 s. One of the main results of the article was also the selection of an appropriate material (Velostat, membrane) that can be used as a sensor for the proposed application solution. The proposed solution can enable communication with the deaf using the finger alphabet, which can be used to spell single words or the most important key words.

## 1. Introduction

According to the report from the Deaf Expert Group on behalf of the Commissioner for Human Rights [1], 850 thousand people in Poland have a hearing disorder, and approximately 100 thousand people use Polish sign language. It is a natural way of communication for the deaf, for whom the Polish language is foreign. According to the report, people with hearing disorders face exclusion resulting from the problem of communication.

Up until now, translating services for the deaf existed in the form of computer software that translated sign language. Such a solution is problematic. There is scarce availability of the service in public and private locations, and access to it is limited to specific ranges of time [2]. This method can also affect person’s comfort and focus because their communication is recorded.

The aim of the work introduced in this article is to recognize the changes made by the user’s hand when showing expressions in sign language. The communication device should not be a burden for the user, but instead, it should be as inconspicuous as possible. The glove form of the device is comfortable. It does not reduce the range of motion, nor does it have an impact on a person’s well-being.

The electronic glove is used as an interface connecting people who encounter problems when communicating in different languages (sign language and spoken language). However, there are other ways to use the device. It can also be used as a keyboard, with the addition of a speaking synthesizer to help people who lack the ability to speak.

Sign language is based on hand movement and finger bending. The key to understand sign language is to know the correspondence of finger states and hand movements to words and phrases. The crucial elements of the device are sensors which recognize the bend level and movement appearance (with precise direction) of the fingers and hands. Each spoken language has its own sign language, and researchers try to find the best solutions to communicate with the deaf [3,4].

So far, the tracking of human body movements, also characteristic of sign language, is carried out with the use of optical systems, which most often use infrared transmitters and receivers [5] To analyze human posture and gestures with the use of optical systems and images, it is necessary to extract human posture features, and subsequently identify and classify them. These studies are related to human physiology, digital image processing, and pattern recognition. For this purpose, neural networks [6,7,8,9] or artificial intelligence [10] are often used. Changes in the electromagnetic field [11], the system of body-worn sensors [12], deep learning [13], or the Fourier decomposition method [14] are also used.

To analyze hand movements, Timothy F. O’Connor’s group [15] developed a glove that uses stress sensors based on a piezoresistive composite composed of carbon particles embedded in a fluoroelastomer. Nine sensors (two on each fingers and one on the thumb) are placed on a leather sports glove. The sensors were connected to the glove using an elastomeric adhesive. The electric circuits are composed of a conductive thread made of stainless steel. The signals are collected in an electronic circuit located on the back of the hand, and then are transmitted via Bluetooth to an external device, where it is recognized and classified as a suitable symbol. The glove was used as a prototype of a device enabling the wireless translation of 26 letters of the American Sign Language alphabet which were processed into text displayed on a computer screen.

Scientists from the University of California attempted to create an intelligent glove for the communication of the deaf with society using modern technological solutions, not only in the field of wearable electronics, but also in the area of computer science. Developed by the Zhou group [15], the pair of gloves consisted of an array of thin, stretchable sensors made of electrically conductive yarn on each finger. The deaf communicate with society by arranging their fingers in the proper way and making a hand movement. The purpose of the sensors is to detect the correct positioning of the fingers and hand movements. Due to the change in the electrical parameters of the yarn from which the sensors are made, a change in electrical signals is observed, which in turn is translated into the identification of individual letters, words, numbers, and even whole phrases [16]. Due to the fact that facial expressions are part of American sign language, self-adhesive sensors were placed on the volunteers’ faces, which, also due to changes in pressure, transmitted correspondingly variable electrical signals to the microcontroller. All transmitted signals were recognized and translated into appropriate characters in a dedicated application [16]. However, the proposed system translates only American Sign Language; it cannot serve, for example, British people due to pronunciation difficulties, despite a similar language [17]. Each native language has its own sign language [4,18,19,20,21]. There are some attempts presented in the literature to construct an intelligent glove that aids in communication with the deaf, but they are not based on the element of textronics [22,23,24]. Textronics—the connection between materials, textiles, electronics, and computer science—is a field of interest for many areas, e.g., electromagnetic field shielding [25], rehabilitation [26], and even bacteria detection [27]. Usually, the thin conducting films are created by the process of magnetron sputtering [28], ink-jet printing [29], or physical vacuum deposition [26].

The authors of this paper present an innovative solution of a glove equipped with bend sensors and an accelerator, which, in cooperation with dedicated software, can serve as a translator of Polish sign language. The proposed flexible technical solutions in the field of textronics do not require the construction of a glove that translates sign language from the production stage. Instead, they enable the adaptation of any type of glove by attaching bend sensors to the inner surface of the material of the existing glove. The developed software that analyzes the data obtained from bend sensors allows for the reading of information in Polish; however, it can be adapted to support any other language. The novelty of this paper is the sensors’ construction on the flexible textile composite substrates as thin electroconductive layers.

## 2. Materials and Methods

Bending level can be easily specified using bend sensors. The sensor works as a potentiometer. It changes its resistance with the component’s bending angle. Bending sensors are available ready-made for purchase. However, they can be easily manufactured using a conductive core. Self-made components are cheaper, flexible along the entire length, and can be easily adjusted to the project. Considering the advantages, self-made bending sensors are used in this project.

All sensors are built based using materials with good electrically conductive properties.

### 2.1. Materials

Among tested materials, the commercial Velostat, also known by the name Linqstat, was used as a reference material. It is a material developed by Custom Materials and is currently a registered trademark of Desco Industries. It is a polymer film (polyolefin) coated with carbon black with very good electrically conductive properties [30]. It is used, inter alia, as a shield for electrostatic discharges [31] or as an element of electrical circuits in “smart” shoes that light up while walking. The phenomenon of resistance changes under pressure or bending is also used. Its surface resistivity is less than 31 kΩ/sq.

The next test materials were Cordura and a Goretex membrane. Cordura is developed by DuPont and is a brand of fabrics showing high resistance to mechanical damage. It is made of polyamide fibers reinforced with a polyurethane coating. The Goretex membrane, on the other hand, is a more flexible material than Cordura, made of foam Teflon fibers covered with a thin layer of nanofibers [32]. Due to the fact that the fibers are covered with layers, ensuring the evenness of the substrate, it is possible to apply a thin electrically conductive layer on the surface of these materials, with a surface resistance on the order of single Ω/sq in the physical vacuum deposition process. A silver layer with a high conductivity value characteristic for this type of metal (γ = 62.5 m/Ω·mm^2^) was applied to the selected materials. The metal evaporation process from the resistive tungsten source lasted 5 min and was carried out on a Classic 250 vacuum stand of the Pfeiffer Vacuum system. The initial vacuum was 5 × 10^−5^ mbar. The silver was delivered and guaranteed by Mennica Metale Ltd. (Warsaw, Poland). The surface material was placed 6 cm from the source of evaporation. The composite textile materials serving as the substrate for the electrically conductive layers were conditioned at room temperature and 55% humidity for several hours prior to the vacuum deposition process. The electrode geometry can be modified using laser ablation of the applied metallic layer [33]. Because some defects can occur during the production process [34], influencing the resistance of the sensor, hot spots can occur [35]; therefore, the validation of the created film can be achieved using optical coherence tomography [36], or optical microscopy.

**Figure 1:**
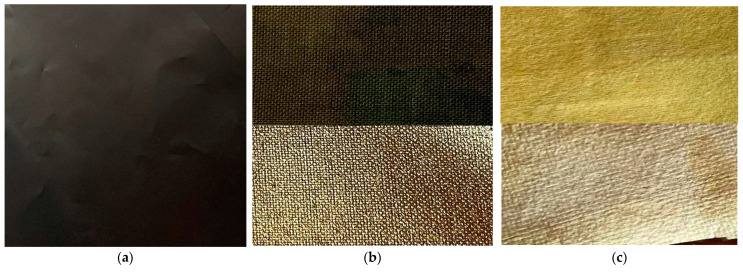
The photo of two sides of the materials (front and back) used for the research: (**a**) Velostat, (**b**) Cordura, (**c**) Goretex membrane.

Photos of the surfaces of the materials used for creating the sensors are presented in Figure 1.

**Figure 2:**
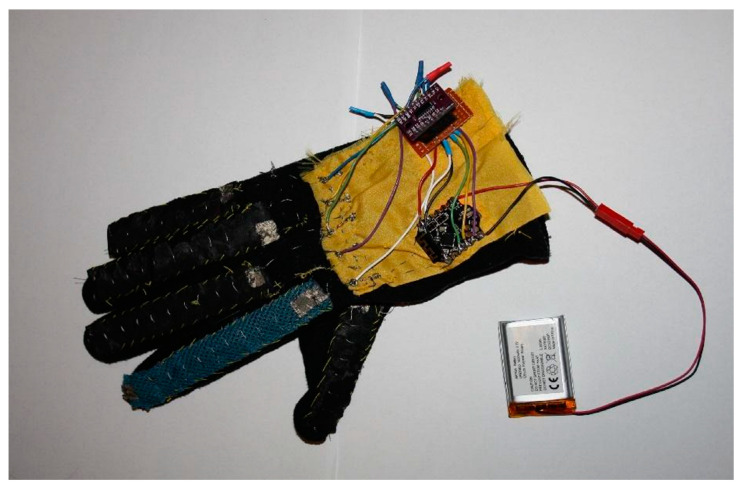
Prototype of a glove for translating sign language.

A photo of a glove prototype is presented in Figure 2. The photograph shows an example of the bend sensors created from electroconductive material, placed on each finger, and also the microcontroller, multiplexer, and power supply system. The microcontroller used in the prototype is a Bluno Beetle from DFRobot (Shanghai, China).

**Figure 3:**
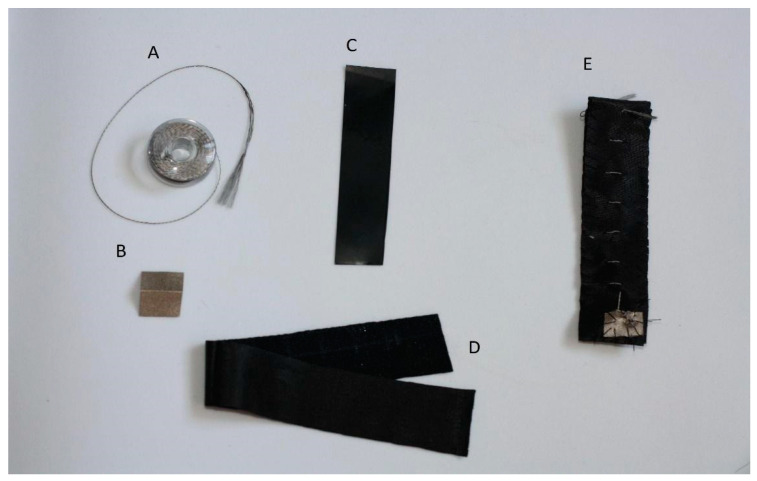
The bend sensor (**E**) and its components—conductive thread (**A**) and material (**B**); Velostat (**C**) as the core, and a piece of fabric (**D**).

The components of the bend sensor are presented in Figure 3. The core is placed between two conductive layers that have no contact with each other. The conductive layers are constructed by making stitches on a piece of fabric. The current flows successively through the first conductive material, the core, and the second conductive material. The resistance of the sensor changes when it is bent.

The resistance range depends on several aspects: the type of material the core is made of, the size of the core, and the size of the gap between the sensor’s layers.

The materials mentioned above were tested as a core of the sensor in order to choose the most suitable fabric. Each sensor with a different core was placed sequentially on a forefinger, and two tests were performed. The first test was to measure the output of the digital signal from the A/D converter when the finger was straight, and the second test measured the output when the finger was maximally bent. The possible range of the signal was 0–1023.

### 2.2. Algorithm

**Figure 4:**
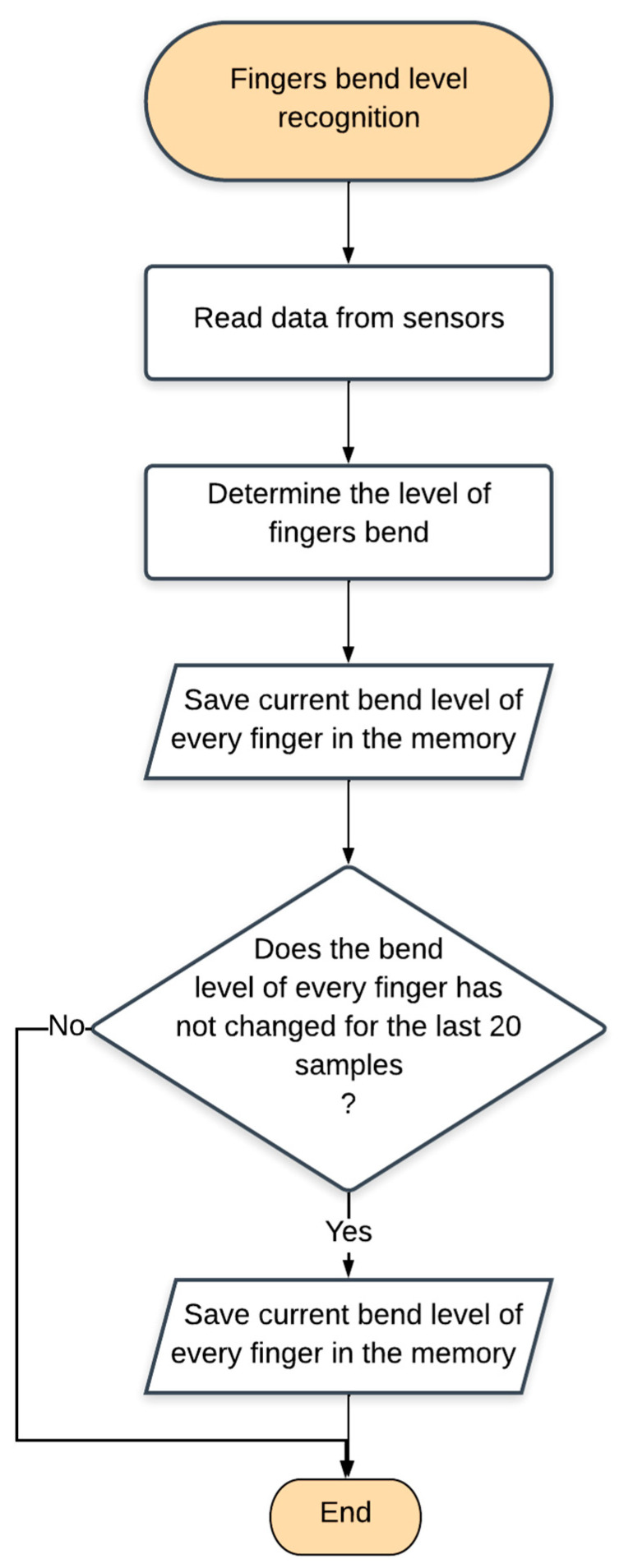
Algorithm showing the recognition of the bend level of the fingers.

Using the introduced parameter, the algorithm to recognize the finger state was developed. After reading the data from the sensors, every finger was been assigned an appropriate bent state, and the results were saved in the memory. After gathering 20 bent states, they were compared. If all of these were identical, the finger was assigned an actual bent state. The process should be run in a thread, performed every 70 ms. The amount of necessary data to be stored in the memory and the thread frequency were determined experimentally, ensuring the correct operation of the device. The described algorithm is presented in Figure 4.

### 2.3. Hand Movement Recognition

Another crucial parameter which must be determined in order to translate sign language is the appearance of movement and its direction. This can be easily detected with an accelerometer. An accelerometer measures accelerations. Acceleration in each direction can be detected using a 3-axis sensor. When the movement starts, the acceleration increases, and when it ends, the acceleration decreases to zero. The 3-axis accelerometer attached to the hand can recognize the movement of the hand, along with the direction of the movement. Hand movements when using sign language are low distance movements, so the probability of uniform motion and therefore, zero acceleration, is small. An accelerometer used in the project is a wearable FLORA accelerometer from Adafruit. It combines a 3-axis accelerometer and magnetometer, using a I2C interface. The working voltage is 3 V.

**Figure 5:**
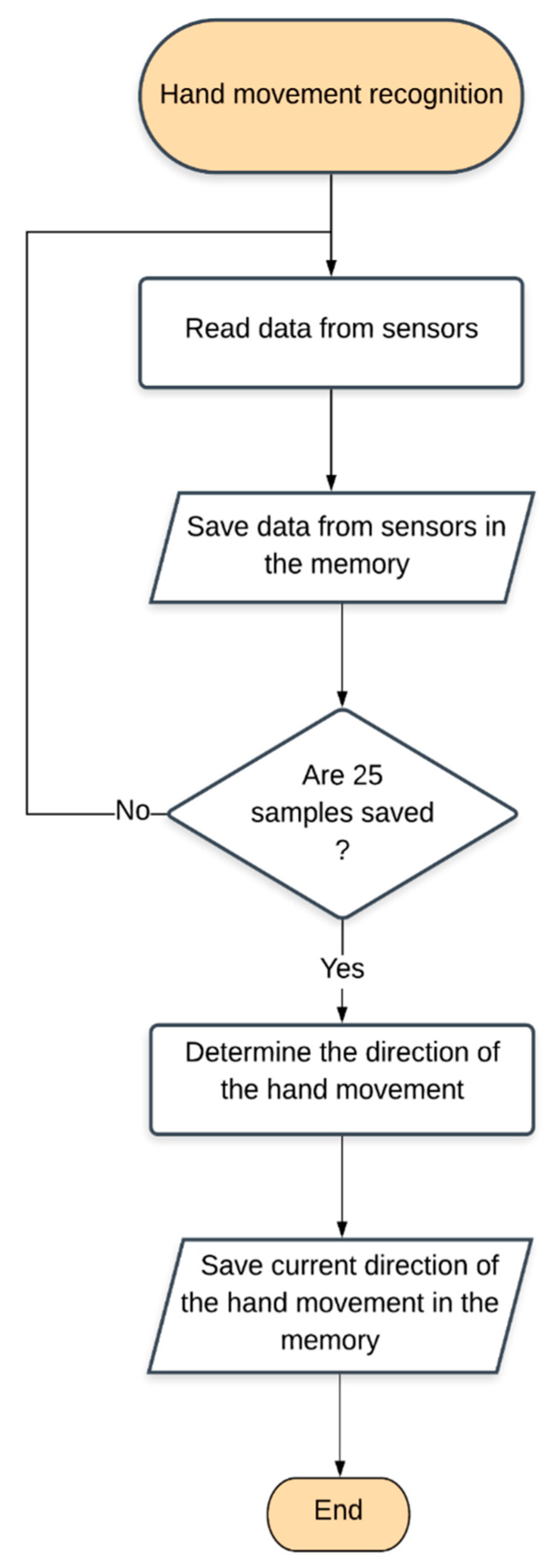
Recognition of hand movement algorithm.

Recognizing the hand movement is executed according to the following algorithm. Data read from an accelerometer is stored in the memory. After storing 25 samples, the direction of the movement is determined and saved as the current hand movement direction. The algorithm ought to be performed every 100 ms. The amount of the necessary acceleration data that have to be stored in the memory and the thread frequency were determined experimentally, ensuring the correct operation of the device (Figure 5).

### 2.4. Sign Identification

**Figure 6:**
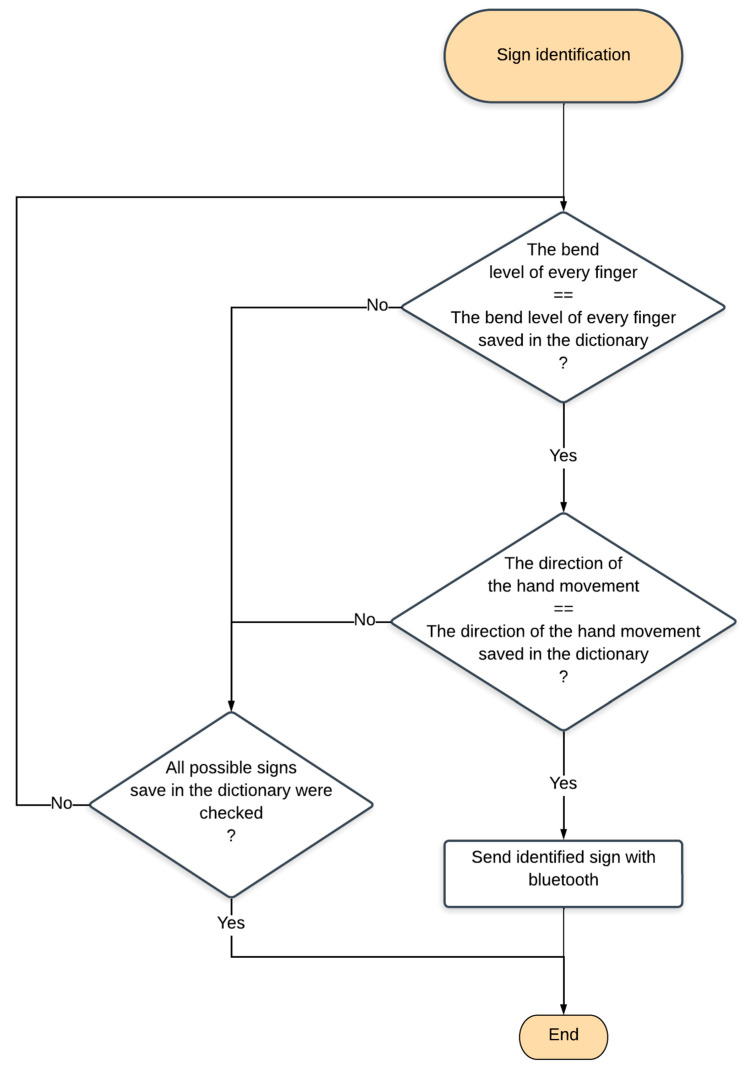
Sign identification algorithm.

The letter identification is processed by software uploaded in the Bluno Beetle microcontroller. The software used to convert electrical to digital signals is the authors’ own software and it is written in C++ language, based on the presented algorithms. The actual state of a hand is compared with values saved in the memory. The action is performed in a thread every 120 ms, with a higher frequency than that of the threads reading the data from the sensors. When the identification is correct, the letter is transmitted via Bluetooth or UART port. The described algorithm is presented in Figure 6.

**Figure 7:**
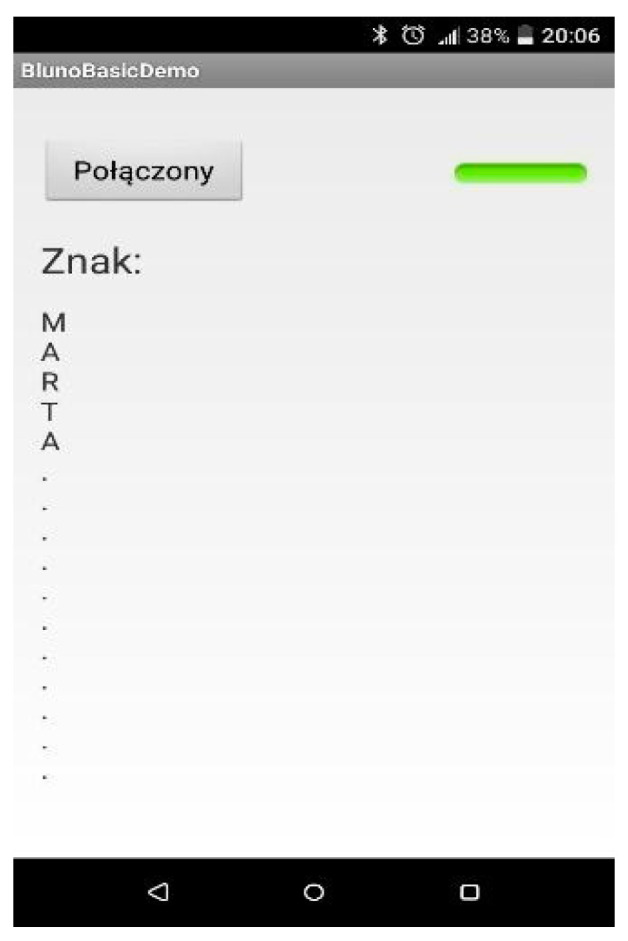
Main screen of the application with a string spelled by the glove.

A mobile application for Android 6.0 was developed. It uses the Bluetooth module of the phone, and after pairing the devices, it prints the letter shown using the created glove (Figure 7).

**Figure 8:**
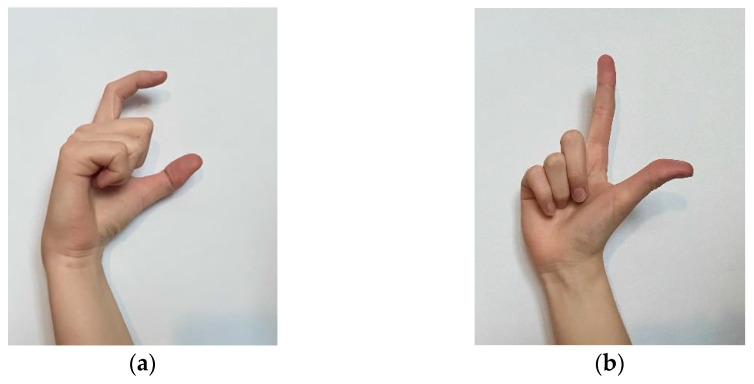
Letter C (**a**) and letter L (**b**) in Polish sign language.

For every letter in a Polish sign language, a set of parameters was determined. Possible combinations are presented in Figure 8. The correct arrangement of the fingers corresponds to each letter. For some letters, the state of a single finger does not have to be specified. The stress of the sensors placed on the fingers is important for distinguishing each letter.

Each finger’s state can be determined by using three scale measurements. The states are: maximally bent, slightly bent (approx. 90°), and straight. The ranges of the sensor’s data values were evaluated experimentally. The sensors were attached to the glove and pinned to the input of the A/D converter. The Min symbol means that the finger should be straightened, Mid means that the finger should be slightly bent, and Max means that the finger should be bent to the maximum. The symbols “−” and “+” in the accelerometer column indicate the need to recognize the hand movements.

The figures below show examples of letters C and L in Polish sign language (Figure 8). The position of the fingers defining a given letter is described in Table 1.

## 3. Results

**Figure 9:**
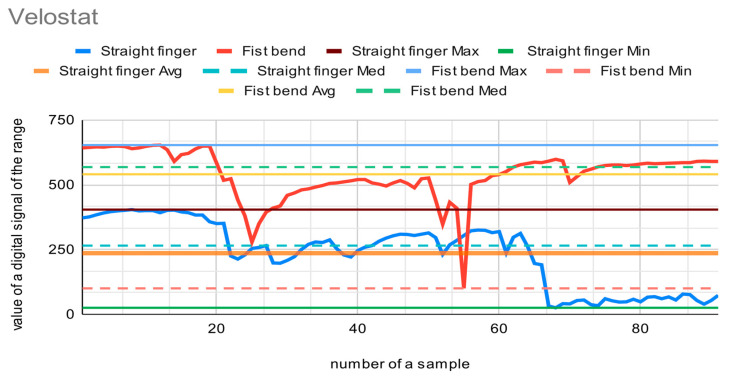
Chart showing samples collected with the Velostat material as a core for the bend sensor.

**Figure 10:**
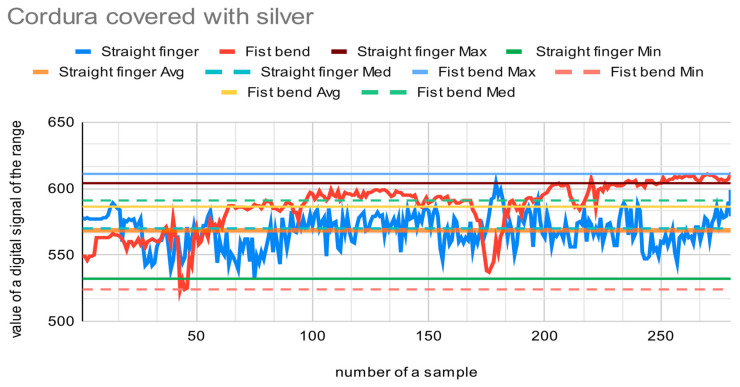
Chart showing samples collected with Cordura covered with silver as a core for the bend sensor.

**Figure 11:**
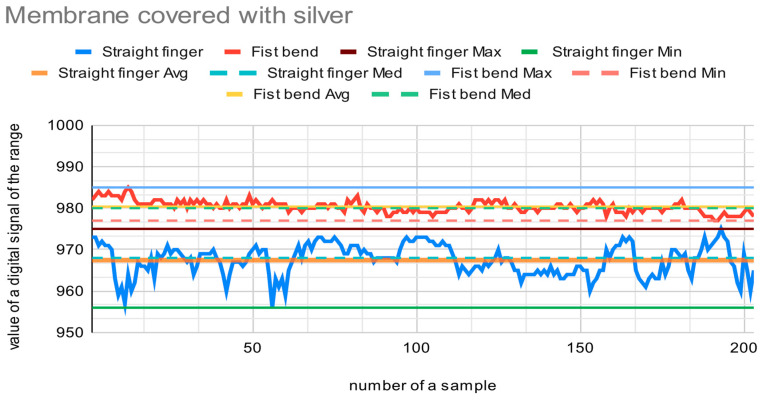
Chart showing samples collected with the Goretex membrane covered with silver material as a core for the bend sensor.

The materials presented in Figure 1 were tested as a core for the sensor in order to choose the most suitable fabric. Each sensor with a different core was placed sequentially on a forefinger and two tests were performed. The first test measured the output of the digital signal from the A/D converter when the finger was straight, and the second test measured the output when the finger was maximally bent. The finger in each position was slightly modified to reflect the natural state of the hand. Fingers recognized as straight may be slightly bent (circa 15 degrees), and fingers bent into a fist may be squeezed more or less firmly. The resolution of the A/D converter used is 10 bits, so it can encode an analog input from 1 to 1023 different levels. Figure 9, Figure 10 and Figure 11 present charts of gathered signals for different core materials. Table 2 shows characteristic values for the measured signals.

After analysis of Velostat’s behavior in the research, it can be observed that the range of the signal is wide, from 25 to 653 levels. The sensor has a resistance range from approx. 9.2 kΩ (straight sensor, lying flat on horizontal surface) to approx. 9 kΩ (sensor bent at an angle of approx. 170 degrees). Two peaks were registered when testing the bend sensor (between the 25th and 55th samples), but it can be assumed that these are individual measurement errors caused by a temporary lack of continuity in the circuit at points joining the conductive thread and the wires.

In the case of the Goretex membrane, the range of the signal is also narrow, from 956 to 983 encoded analog the signal. The sensor has a resistance range from approx. 155 Ω (straight sensor, lying flat on horizontal surface) to approx. 90 Ω (sensor bent at an angle of approx. 170 degrees). The bending resistance of the thin layer created on the membrane and Cordura is described in the previous paper [37].

Cordura is the most rigid material among all those tested. Because of this feature of the material, the signal values do not differ when the finger is bent or straight. The resistance of the sensor fluctuates between 200 Ω (straight sensor, lying flat on horizontal surface) to approx. 208 Ω (sensor bent at an angle of approx. 170 degrees). The sensor does not work as expected with this material; therefore, it cannot be used as a core for the sensor.

The best material among those tested is the Velostat. The range of the signal is wide, as is the resistance. It gives the most accurate signal values. This is caused by the additional aspect that when bending, the sensor with the Velostat membrane causes pressure on the membrane, which leads to an additional change in its resistance. The Velostat is also durable. The membrane covered with silver provides accurate signal values as well. However, the range of the signal is narrow. On the basis of this test, the Velostat membrane was chosen as a core for the bending sensor.

Every finger of the created glove was equipped with one bend sensor in order to measure the bent angle of the respective finger.

**Figure 12:**
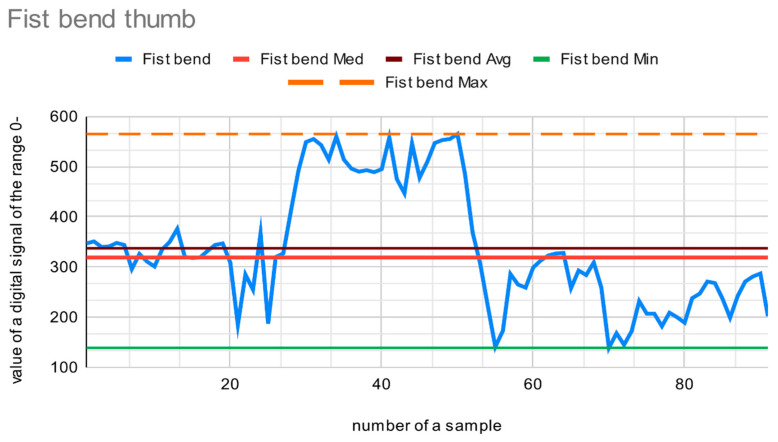
Data from the sensor on a maximally bent thumb.

Two measurements were performed: first, when the fingers were straight, and second, when the fingers were maximally bent. During the process, the fingers were moved slightly—into a fist, tightly or slightly clenched, and then relaxed—with the fingers fully and loosely straightened. This was completed to unify the bend level of the measured fingers. It was assumed that the finger is classified as straight when it is bent in an angle up of 0–20° (the bend angle of a fully straightened finger is 0°). The finger is considered to be maximally bent when the bend angle is between 160–190°. This ensures the procurement of a wide range of sensor data from each bending state. The described test was performed once on a single person. The resulting ranges were adjusted during manual testing of the glove, in which the recognition of individual letters was checked. The possible data values were in the range of the 0 to 1023 level. Every finger has its own data values range. Figure 12 displays values gathered for the thumb when maximally bent. The minimal value from the sensor is 139. The maximum is 568. The average is 339 and the median 319. The median and average values are considered to provide perspective regarding the most probable and the most common signal values that may appear.

**Figure 13:**
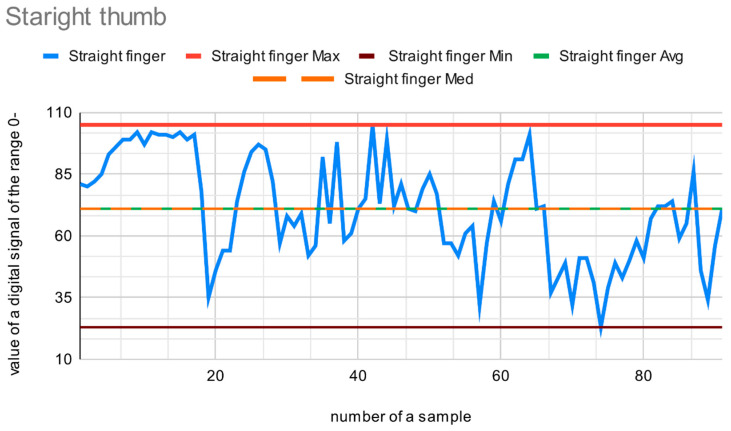
Data from the sensor on a straight thumb.

Figure 13 shows values from the sensor on a straight thumb. The maximum value is 105, and the minimum is 23. The average and median are 71.

The range of values between the maximum value, when the finger is straight, and the minimal value, when the finger is maximally bent, is a range of values for the third possible state of the finger—slightly bend (approx. 90°).

The additional plots of data received for other fingers and their states are found in Appendix A.

Table 3 presents the ranges of signal levels for every finger in the three possible bend states.

Tests evaluating the correctness of the interpretation of individual letters were carried out by the authors. As a part of testing the glove, a fragment of a poem consisting of 520 letters was translated, and the effectiveness was assessed (the percentage of correctness of the translation of the text) Depending on the signing ability (in particular, the speed of change of the individual hand settings), the correctness of letter recognition was 86.5%. The recognition time of each sign was within approximately 3 s.

## 4. Discussion

The testing phase was focused on recognizing a fingers’ bent states. In Table 1, the blue color indicates the letters that are not easily identified. For example, the letters E, M and S, O are shown in a similar way. They differ from each other only with a state of a single finger or hand orientation; whereas, the letter G is shown as a finger movement. The identification of this letter should be performed in an alternative way to the rest of letters.

Another issue is that any force acting on a bend sensor is interpreted as a finger bending. This occurs when adjacent fingers are in extreme positions, e.g., for the letter L, the forefinger is straight and the middle finger is maximally bent. The membranes inside the sensors are stretched, which provides an incorrect data value for the straight finger. Another example is the letters R and T, where the fingers have contact at the location of the sensors. Finger pressure causes a force on the sensors, resulting in incorrect letters identification.

Another issue with incorrect letter identification is connected with the glove’s cut. The glove should be well fitted to the hand in order to provide correct and repeated letter identification.

Based on the tests performed, improvements to the concept of the device can be introduced. Adding an additional bending sensor on the forefinger—one sensor for measuring the bend level between the finger and the palm, and the other in the middle of the finger—can improve the repetitiveness of letter identification, defining the bend angle of the finger itself. This provides better recognition between different states of the finger.

Another modification is the introduction of a new parameter defining hand orientation. In case of the letters M, E, and U, this is a crucial aspect.

Experiments have shown that the system can be tuned to the individual characteristics of the person, and the correctness of letter detection can be increased above 86.5%, even in the case of the most difficult to recognize letters.

In the literature, for all solutions using gloves, the analogue signal is converted to a digital one with the use of various sensors. The use of thin electroconductive layers for this type of solution is a new approach not previously found in the literature. The possibility of attaching a flexible sensor to any type of glove at any stage of its use is a value added to the presented solution. The authors indicated materials that can be used for this type of application.

## 5. Conclusions

The process of manufacturing and testing a prototype of a glove for translating sign language was introduced. It can be used as to aid communication between people using sign language and those who do not know it. The application can be an alternative for a keyboard. The device lets the user input letters using a mobile device or a computer. The process of letter identification is not fully precise. The arrangement of the fingers when pointing to a specific letter is always the same; however, even slight changes in the angle of flexion may affect the identification of the information. At the present stage of research, the influence of mechanical stress on the durability of the thin conductive layers should also be taken into account. Extending the life of textronic sensors is a challenge facing current science. In order to increase the correctness of operation, improvements should be applied. One of the improvements is adding an additional bending sensor on the forefinger in order to distinguish the bend level of a bent finger itself and the bend level of the finger in relation to the palm. Another improvement is implementing an additional parameter defining the hand orientation. It is worth conducting further research to achieve the increased resistance of thin electroconductive layers to bending stress. The possibility of using materials coated with thin electrically conductive layers in a signing glove application is a difficult challenge due to the mechanical strength of such layers. The analysis of their electrical properties and the selection of substrates for the production of thin-film sensors allowed for the construction of a glove that was used to interpret over 500 signs, with a correctness at the level of 86.5%. The obtained results are very satisfactory and promising. Constructing a glove that will be a bridge between the deaf and hearing population constitutes a tool that will improve communication between these two groups.

## Figures and Tables

**Table 1:** Combinations of parameters that must be satisfied for every letter. Max—finger bent maximally; Mid—finger slightly bent; Min—straight finger. The accelerometer column shows whether usage of the accelerometer is necessary for the finger. Blue color indicates the letters that are not easily identified (more in Section 4, Discussion).

Letter	Thumb	Forefinger	Middle Finger	Ring Finger	Little Finger	Accelerometer
A	Max/Mid/Min	Max	Max	Max	Max	−
Ą	Max/Mid/Min	Max	Max	Max	Max	+
B	Max/Mid/Min	Min	Min	Min	Min	−
C	Mid	Mid	Max	Max	Max	−
Ć	Mid	Mid	Max	Max	Max	+
CH	Mid	Mid	Max	Max	Max	+
CZ	Mid	Mid	Max	Max	Max	+
D	Max/Mid/Min	Min	Max	Max	Max	+
E	Mid	Mid	Mid	Mid	Mid	−
F	Min	Mid	Min	Min	Min	+
G	Mid	Min	Max/Mid/Min	Max/Mid/Min	Min	−
H	Max	Mid	Mid	Max	Max	+
I	Max/Mid/Min	Max	Max	Max	Min	−
J	Max/Mid/Min	Max	Max	Max	Min	+
K	Min	Min	Min	Max	Max	+
L	Min	Min	Max	Max	Max	−
M	Min	Mid	Mid	Mid	Mid	−
N	Mid	Mid	Mid	Max	Max	−
O	Mid	Mid	Min	Min	Min	−
P	Mid	Mid	Max	Max	Max	−
R	Mid/Min	Mid	Min	Max	Max	−
RZ	Mid/Min	Mid	Min	Max	Max	+
S	Mid	Min	Min	Min	Min	−
Ś	Mid	Min	Min	Min	Min	+
SZ	Mid	Min	Min	Min	Min	+
T	Max	Mid	Min	Min	Min	−
U	Max	Min	Min	Max	Max	−
W	Max	Min	Min	Min	Max	−
Y	Max	Min	Max	Max	Min	−
Z	Max/Mid/Min	Min	Max	Max	Max	−
Ż	Max/Mid/Min	Min	Max	Max	Max	+
Ź	Max/Mid/Min	Min	Max	Max	Max	+

**Table 2:** Encoded analog signal at levels from 0 to 1023. S—straight finger; B—bent finger.

Material		Maximum Value	Minimum Value	Average Value	Median Value
Velostat	S	404	25	236	265
	B	653	100	540	568
Cordura	S	604	532	569	570
	B	611	524	586	591
Goretex membrane	S	975	956	967	968
	B	983	977	980	980

**Table 3:** Range of digital signal values for every bend state.

Finger	Straight	Slightly Bent		Maximally Bent
Thumb	less than 100	101	199	over 200
Forefinger 1	less than 230	231	379	over 380
Middle finger 2	less than 310	311	379	over 380
Ring finger 3	less than 230	231	369	over 370
Little finger 4	less than 100	101	199	over 200

**Figure A1:**
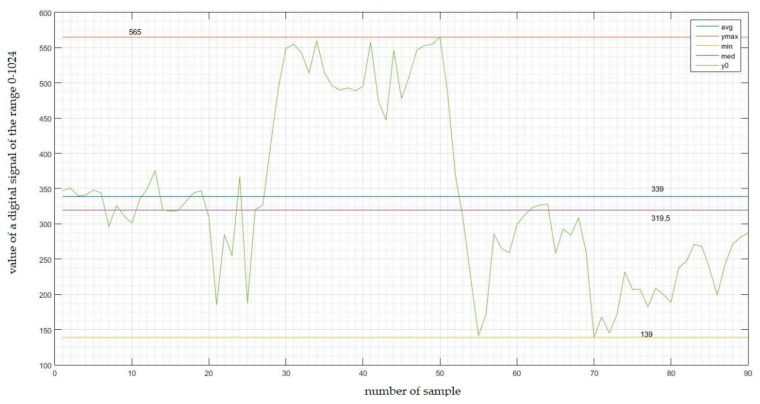
Data from the sensor on a maximally bent thumb.

**Figure A2:**
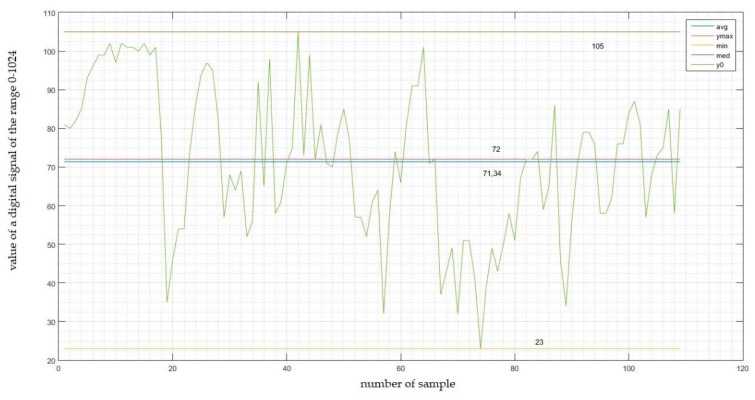
Data from the sensor on a straight thumb.

**Figure A3:**
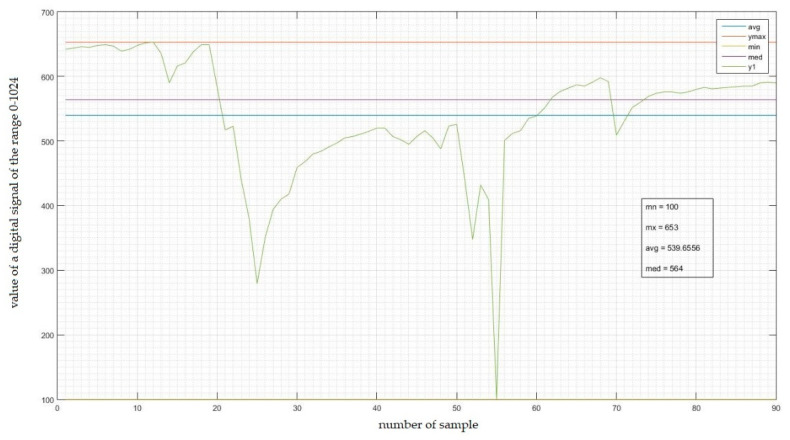
Data from the sensor on a maximally bent forefinger.

**Figure A4:**
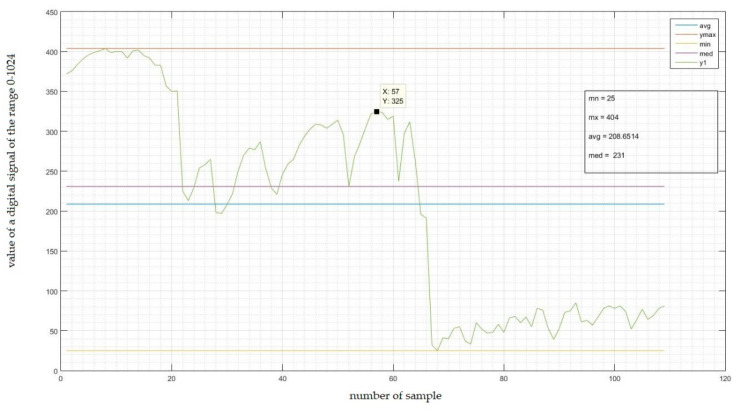
Data from the sensor on a straight forefinger.

**Figure A5:**
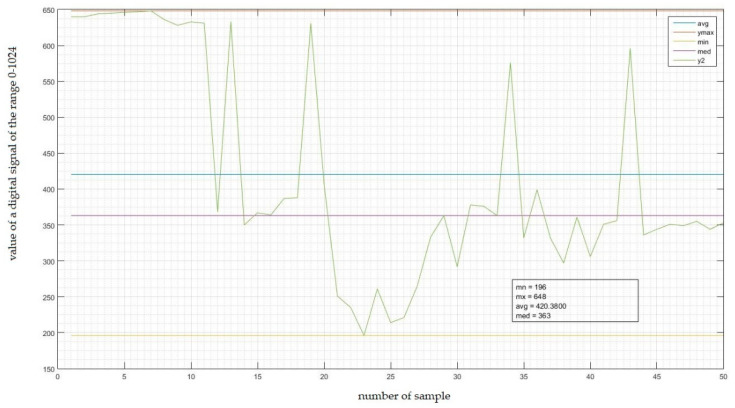
Data from the sensor on a maximally bent middle finger.

**Figure A6:**
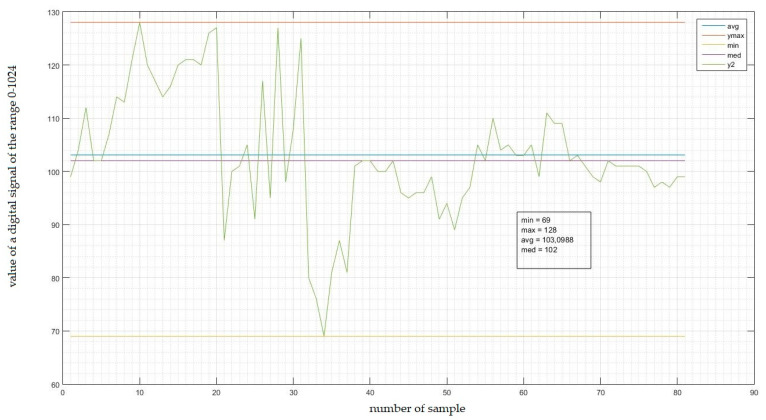
Data from the sensor on a straight middle finger.

**Figure A7:**
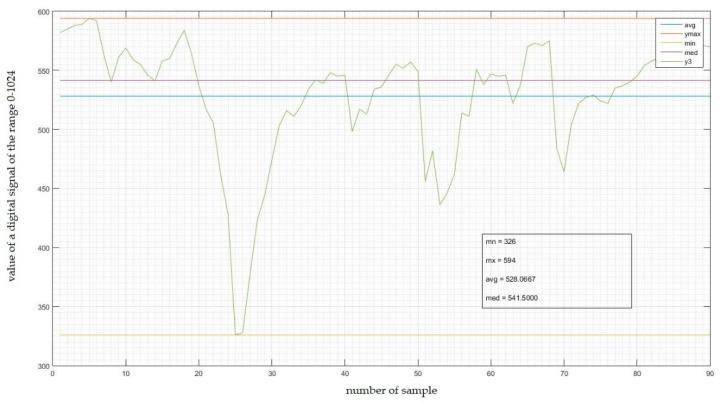
Data from the sensor on a maximally bent ring finger.

**Figure A8:**
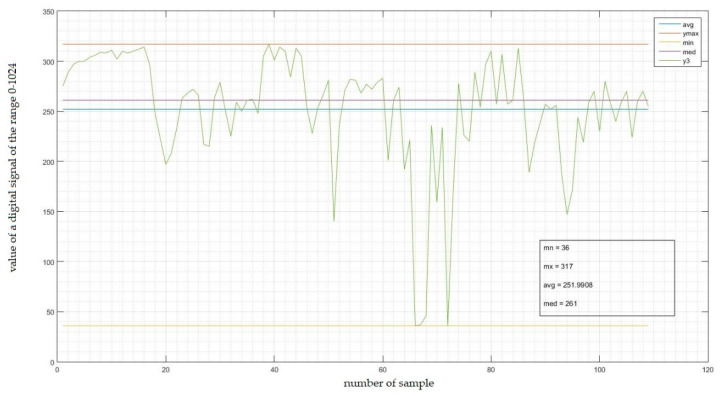
Data from the sensor on a straight ring finger.

**Figure A9:**
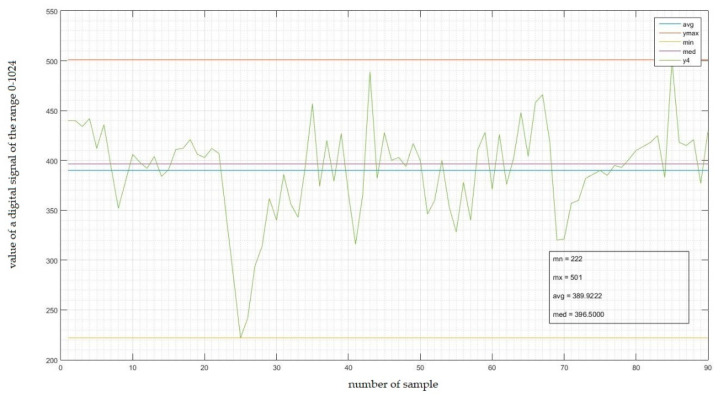
Data from the sensor on a maximally bent little finger.

**Figure A10:**
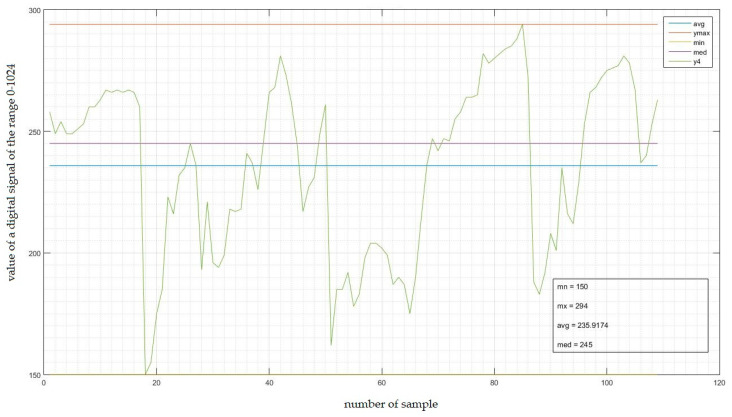
Data from the sensor on a straight little finger.

## Data Availability

Not applicable.

## Appendix A

Some additional figures showing representative data acquired from the sensors placed on the fingers of the glove are presented in Figure A1, Figure A2, Figure A3, Figure A4, Figure A5, Figure A6, Figure A7, Figure A8, Figure A9 and Figure A10.
